# The Preoperative Supplementation With Vitamin D Attenuated Pain Intensity and Reduced the Level of Pro-inflammatory Markers in Patients After Posterior Lumbar Interbody Fusion

**DOI:** 10.3389/fphar.2019.00527

**Published:** 2019-05-22

**Authors:** Katarzyna Krasowska, Wojciech Skrobot, Ewelina Liedtke, Piotr Sawicki, Damian Jozef Flis, Katarzyna Patrycja Dzik, Witold Libionka, Wojciech Kloc, Jan Jacek Kaczor

**Affiliations:** ^1^Department of Kinesiology, Gdansk University of Physical Education and Sport, Gdańsk, Poland; ^2^Department of Sport, Gdansk University of Physical Education and Sport, Gdańsk, Poland; ^3^Department of Bioenergetics and Nutrition, Gdansk University of Physical Education and Sport, Gdańsk, Poland; ^4^Department of Neurobiology of Muscle, Gdansk University of Physical Education and Sport, Gdańsk, Poland; ^5^Department of Neurosurgery, Copernicus Hospital Gdansk, Gdańsk, Poland; ^6^Department of Neurology and Neurosurgery, University of Warmia and Mazury in Olsztyn, Olsztyn, Poland

**Keywords:** vitamin D, inflammatory cytokines, low back pain, VAS, early rehabilitation

## Abstract

The aim of this experimental study was to assess whether 5 weeks of preoperative supplementation with vitamin D affects the intensity of pain and the level of inflammatory markers in patients undergoing posterior lumbar interbody fusion (PLIF) followed by rehabilitation. 42 patients were divided, by double-blind randomization, into two groups: supplemented (SUPL) vitamin D (3200 IU dose of vitamin D/day for 5 weeks) and placebo group (PL) treated with vegetable oil. The 10-week program of early rehabilitation (3 times a week) was initiated 4 weeks following PLIF. Measurements of serum 25(OH)D_3_ and CRP, IL-6, TNF-α, and IL-10 were performed. Pain intensity was measured using VAS. After supplementation with vitamin D serum, the concentration of 25(OH)D_3_ significantly increased in the SUPL group (^∗^*p* < 0.005) and was significantly higher as compared to the PL group (^∗^*p* < 0.001). A significant reduction in pain intensity was observed 4 weeks after surgery and after rehabilitation in both groups. In the SUPL group, serum CRP and IL-6 concentration significantly decreased after rehabilitation, compared with the postsurgical level (^a^*p* < 0.04). The level of TNF-α was significantly lower after rehabilitation only in the supplemented group (^∗^*p* < 0.02). There were no significant changes in the IL-10 level in both groups during the study. Our data indicate that supplementation with vitamin D may reduce systemic inflammation and when combined with surgery and early postsurgical rehabilitation, it may decrease the intensity of pain in LBP patients undergoing PLIF. Data indicate that LBP patients undergoing spine surgery should use vitamin D perioperatively as a supplement.

## Introduction

Musculoskeletal pain including low back pain (LBP) is very common, affecting from 10% (Brooks, 2006; McBeth and Jones, 2007) to 33% (Croft et al., 1993) of the general population (Al-Jarallah et al., 2013). Approximately 50–80% of people have at least one episode of LBP during their lifetime (Hoy et al., 2010, 2014) and symptoms often recur (Steffens et al., 2016). LBP affects people of all ages and according to the 2010 Global Burden of Disease Study, is amongst the top 10 diseases that account for the highest number of Disability-Adjusted Life Years worldwide (Vos et al., 2012). A lot of risk factors have been identified, such as age, gender, overweight, non-neutral work postures, smoking, etc., (Colombini et al., 2014), which are linked with spinal damage and severe pain. The degeneration of the intervertebral disk is one of the primary causes (Borenstein et al., 2001). The pathology of intervertebral foramens or the dysfunction of ligaments, when refractory to conservative treatment or instable, are subject to surgery. Therefore, postoperative rehabilitation provides a quick recovery, which is needed to rebuild the system of stabilizing muscles and to prevent further instability injuries. In addition, postoperative rehabilitation serves to relearn ergonomics and to return to daily activities. Hence the quest for methods that would speed up convalescence.

Vitamin D is a fat-soluble vitamin that is synthesized from a precursor in the skin after exposure to solar ultraviolet B radiation and also from dietary sources. According to the Endocrine Society Clinical Practice Guideline on the Prevention of Vitamin D Deficiency, the concentration of serum 25(OH)D_3_ from 21 to 29 ng/mL (52.5–72.5 nmol/L) is insufficient and a level lower than 20 ng/mL (50 nmol/L) is considered as deficient (Holick et al., 2011; Pludowski et al., 2018). Sufficient serum vitamin D concentrations may be protective against a range of disease states, including cancer, cardiovascular disease, diabetes and multiple sclerosis, and may enhance the immune system (Schwalfenberg, 2007). Vitamin D deficiency is associated with diffuse muscle pain and muscle weakness (Holick, 2007) predominantly in the proximal muscle groups. A recent study shows that the deficiency of vitamin D has been linked to myopathy, muscle pain, and impaired gait (Girgis et al., 2013). Moreover, it has also been demonstrated in people with serum deficiency of vitamin D that supplementation with vitamin D may improve muscle strength (Bischoff-Ferrari, 2012).

The vitamin D receptor (VDR) has been identified in muscle tissue and could explain the association with muscle weakness and regional pain disorders such as low back pain (McBeth et al., 2010). The presence of VDR is evident in skeletal muscle (Dahlquist et al., 2015) and also in intervertebral disk cells, more specifically in the nucleus pulposus and annulus fibrosus cells, which constitute the two different major parts of the intervertebral disk (Colombini et al., 2012). Moreover, recent studies have also reported that a sufficient serum concentration of vitamin D may protect against the reduction of VDR content, oxidative stress, and muscle atrophy (Bang et al., 2018; Dzik et al., 2018).

Vitamin D plays a major role in the modulation of inflammatory pathways by increasing anti-inflammatory and decreasing pro-inflammatory cytokines (D’Ambrosio et al., 1998) and has immunosuppressive properties (Kruit and Zanen, 2016). A higher serum concentration of C-reactive protein (CRP), a marker of inflammation, has been observed when the synthesis of vitamin D was limited, especially in winter (Tao et al., 2015; Aglipay et al., 2017). Other studies have shown that a long-term vitamin D deficiency is associated with an attenuation of the immune system and chronic inflammation (Mangin et al., 2014; Jackson et al., 2016). Furthermore, it has been presented that the level of CRP is higher in patients with a deficiency, as compared to patients with a sufficient level of vitamin D (Laird et al., 2014). Therefore, based on recent publications, we assume that a normalized serum concentration of vitamin D will have a positive impact on the immune system and intensity of pain in LBP patients. Moreover, 1α,25-(OH)_2_D_3_ effectively up-regulates the synthesis of the anti-inflammatory cytokine, interleukin 10 (IL-10) and induces IL-10 receptor expression *in vitro* (Canning et al., 2001). The main aim of this study was to assess, whether 5 weeks of supplementation with vitamin D will attenuate the intensity of pain and reduce the serum level of pro-inflammatory cytokines in patients directed for PLIF surgery. We hypothesized that LBP patients supplemented with vitamin D would have a better and faster recovery with reduced pain intensity.

## Materials and Methods

### Subjects

**FIGURE 1:**
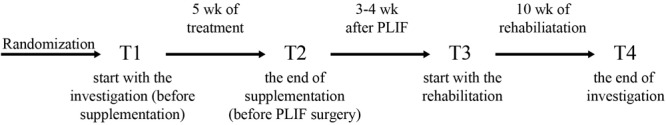
The timelines of the program.

The study was a double-blinded, randomized controlled trial. The study was approved by the local institutional Bioethical Committee in Gdansk (No. NKBBN/120/2012), conformed with the Declaration of Helsinki, and was registered as a Clinical Trial NCT03417700. Written informed consent was obtained from the participants of this study. The study included patients aged 33–65 year qualified by a neurosurgeon for lumbar spine surgery utilizing static or dynamic implants (posterior lumbar interbody fusion, PLIF). Qualification criteria for PLIF surgery included: classification procedures segmental instability, secondary to segmental spinal pathology, discopathy, permanent back pain in patients after non-surgical treatments without improving back pain, resulting in functional disability for at least 6 months, and no history of previous spinal surgery. The exclusion criteria were the following: patients beyond the age range, a previous lumbar spinal operation, spinal tumors, new fractures, an inability to follow the rehabilitation protocol, and a failure to perform daily activities due to comorbidities (e.g., Parkinson disease, patients with mental disorders such as dementia, and psychiatric disorders), and a missed follow-up visit. All the patients used perioperative antibiotics, non-steroidal anti-inflammatory drugs (NSAIDs), perfalgan, and tramadol for the same time duration. 39 out of 42 enrolled patients were included in the study, based on the qualification and exclusion criteria. 3 patients who resigned at the beginning of the study were excluded from further analyses. Three patients smoked and none of the qualified patients drank alcohol while the project was running. Fifteen patients were noted to have hypertension after being examined by a medical doctor. The patients were randomly divided into two groups: the placebo group (PL) was supplemented with vegetable oil, whereas the supplemented group (SUPL) obtained 3200 IU of vitamin D/day for 5 weeks (Vigantol, Merck). The characteristics of patients are presented in Table 1. Before the start of supplementation, the patients received dark glass bottles with droppers, in which there was vitamin D or vegetable oil. Patients were required to take 5 drops of vitamin D or placebo once a day. The process of supplementation during these 5 weeks was controlled by a designated person. After 5 weeks of supplementation, patients were directed for the PLIF surgery performed at a single referential center. Three to four weeks after the operation, the patients were assigned to a personal physiotherapist for 10 weeks of early rehabilitation 3 times a week. All the patients underwent ergonomic behavior instructions before PLIF surgery. At the beginning of the first weeks after surgery, patients were encouraged to perform daily activities, mainly a short walk with some self-control without pain. Patients initiated a supervised rehabilitation protocol 3–4 weeks after PLIF surgery and performed the same procedures. The load of the exercises was increased every week. The main aim of the rehabilitation program was to obtain better spine stabilization, through the enhancement and activation of muscles, which are responsible for core stabilization. The timelines of the program are shown in Figure 1. Blood and VAS (Visual Analogue Scale) score was collected from all patients four times: before supplementation (T1), after supplementation (T2), before rehabilitation (T3) and after rehabilitation (T4).

**Table 1:** The characteristics of patients involved in the study.

	Age	BMI
**SUPL**	**41.92 ± 2.97**	**29.15 ± 1.15**
F (*n* = 9)	46.00 ± 5.99	29.06 ± 1.75
M (*n* = 9)	38.43 ± 1.64	29.25 ± 1.62
**PL**	**47.33 ± 2.15**	**28.14 ± 0.51**
F (*n* = 12)	45.75 ± 2.53	27.95 ± 0.75
M (*n* = 9)	49.44 ± 3.77	28.39 ± 0.69
**P**	**Ns**	**Ns**

### Blood Analysis

Blood samples were taken from the antecubital vein into the vacutainer tubes with silica clot activator. After centrifugation at 2000 × *g* for 10 min at 4°C, the serum was collected and stored in aliquots at -80°C until analysis.

Serum 25(OH)D_3_ level was determined using the 25-OH Vitamin D total ELISA kit (DE1971, Demeditec Diagnostics, Germany) according to the manufacturer’s instructions. The intra-assay coefficients of variability (CVs) and inter-assay CVs reported by the manufacturer were 2–8% and 4–9%, respectively. Serum CRP, interleukin-6 (IL-6), tumor necrosis factor alpha (TNF-α), and interleukin-10 (IL-10) concentration were measured using the ELISA kit (DCRP00, HS600B, HSTA00E, and HS100C, respectively: R&D, United States) according to the manufacturer’s instructions. The intra-assay coefficients of variability (CVs) and inter-assay CVs reported by the manufacturer were 2–8% and 4–9%, respectively.

### Severity of Pain

Visual Analogue Scale (VAS) was used to determine the intensity of pain in both groups. At each time point the patients were asked to rate their pain intensity on a 10 cm VAS (from 0 to 10), with 0 indicating no pain and 10 “worst possible pain.”

### Statistical Analyses

Statistical analyses were performed using a software package (Statistica v. 13.0, StatSoft Inc., Tulsa, OK, United States). The results are expressed as the mean ± standard error (SEM). The differences between the means (time points and between-group term) were tested using repeated measurements (RM) ANOVA. If a difference was detected in the ANOVA model, the significant differences were determined using Bonferroni’s *post hoc* test. The results were statistically significant at *p* < 0.05.

## Results

### Vitamin D Levels

**FIGURE 2:**
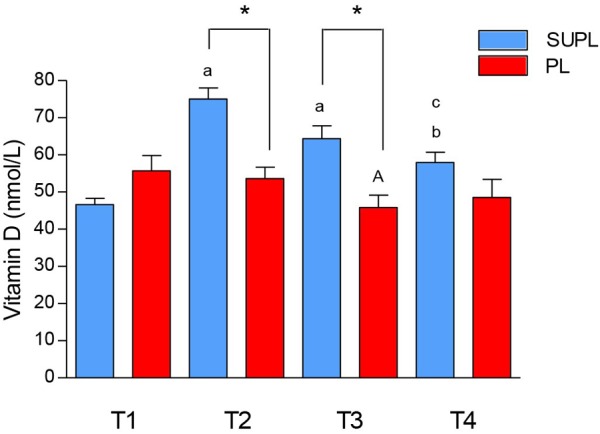
The concentration of vitamin D in serum of LBP patients. Results were expressed as mean ± SEM. SUPL (*n* = 18), PL (*n* = 21), ^∗^*p* < 0.001 – difference between the means between indicated groups. ^a^*p* < 0.005 – difference between the indicated results/mean and T1 SUPL. ^b^*p* < 0.05 – difference between the indicated results/mean and T1 SUPL. ^c^*p* < 0.0002 – difference between the indicated results/mean and T2 SUPL. ^A^*p* < 0.03 – difference between the indicated results/mean and T1 PL.

The initial serum 25(OH)D_3_ concentration in LBP patients was insufficient in both study groups. There was a significant increase in serum 25(OH)D_3_ in the SUPL group after 5 weeks of supplementation with vitamin D (from 46.63 ± 1.69 to 75.03 ± 3.03 nmol/L, ^∗^*p* < 0.02; Figure 2). In the PL group, as expected, supplementation with placebo did not affect serum 25(OH)D_3_ concentration (55.71 ± 4.11 before and 53.62 ± 3.07 nmol/L after treatment). The level of vitamin D in serum was also significantly higher in the SUPL group as compared to the PL group after supplementation (T2; ^∗^*p* < 0.001; Figure 2). The serum level of vitamin D in the PL group significantly decreased after surgery (T1 vs. T3, ^a^*p* < 0.03; Figure 2). In the SUPL group, the serum 25(OH)D_3_ concentration that reached sufficient value after 5 weeks of treatment, remained at the level close to the desired one after surgery and after rehabilitation (T1 vs. T2, T3 and T4; ^a^*p* < 0.005, ^b^*p* < 0.05, respectively; Figure 2). Similar changes were not observed in the PL group in LBP patients.

### Pain Severity

**FIGURE 3:**
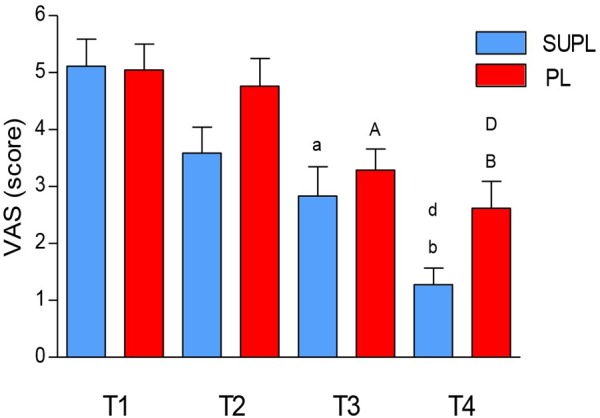
The effect of vitamin D supplementation on VAS. Results were expressed as mean ± SEM, SUPL (*n* = 18), PL (*n* = 21); ^a^*p* < 0.006 – difference between the indicated results/mean and T1 SUPL, ^b^*p* < 0.0001 – difference between the indicated results/mean and T1 SUPL, ^d^*p* < 0.002 – difference between the indicated results/mean and T2 SUPL. ^A^*p* < 0.05 – difference between the indicated results/mean and T1 PL. ^B^*p* < 0.0001 – difference between the indicated results/mean and T1 PL. ^D^*p* < 0.001 – difference between the indicated results/mean and T2 PL.

We did not observe a significant change in pain intensity after 5 weeks of the vitamin D or placebo supplementation. However, there was a significant reduction in the VAS score after surgery and after rehabilitation of LBP patients in both groups (T1 vs. T3 and T4; ^a^*p* < 0.006, ^b^*p* < 0.0001, ^a^*p* < 0.02, ^b^*p* < 0.0001; Figure 3). The degree of pain reduction tended to be higher in the SUPL group as compared to the PL group, both after surgery and rehabilitation (2.83 ± 0.51 vs. 3.29 ± 0.37, T3 and 1.28 ± 0.29 vs. 2.62 ± 0.47, T4; in the SUPL and the PL groups, respectively; Figure 3).

### The Level of Pro and Anti-inflammatory Cytokines

**FIGURE 4:**
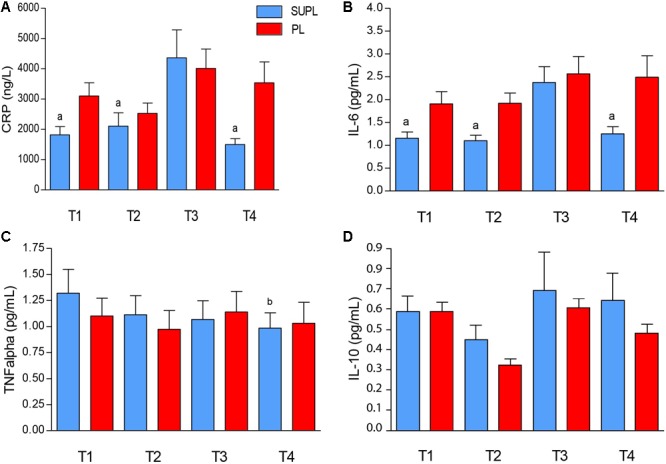
The serum CRP **(A)**, IL-6 **(B)**, TNF- α **(C)**, and IL-10 **(D)** concentrations in LBP patients. Results were expressed as mean ± SEM. SUPL (*n* = 18), PL (*n* = 21); ^∗^*p* < 0.05 – difference between the means indicated groups; ^a^*p* ≤ 0.04 – difference between the indicated results/mean and T3 SUPL, ^b^*p* < 0.02 – difference between the indicated results/mean and T1 SUPL.

The initial serum level of CRP tended to be higher in the PL group as compared to the SUPL group (3102.8 ± 435.7 and 1819.9 ± 271.1 ng/L, respectively; Figure 4A). In both groups, initial serum CRP concentration did not change after vitamin D or placebo supplementation, but increased after surgery. However, significant CRP concentration reduction after rehabilitation was found only in the SUPL group (T3 vs. T4; ^a^*p* < 0.04; Figure 4A). The differences between groups did not reach significance because of a high standard deviation (Figure 4A). The same tendency in the level of IL-6 was observed. At baseline, the serum IL-6 level tended to be higher in the PL group as compared to the SUPL group (1.91 ± 0.26 and 1.15 ± 0.13 pg/mL, respectively; Figure 4B). There were no changes in serum IL-6 level after supplementation, but we observed a significant increase in this marker after PLIF surgery (2.38 ± 0.35 pg/mL for the SUPL group; Figure 4B). Interestingly, a reduction in the level of IL-6 after the rehabilitation program was found only in the SUPL group (1.25 ± 0.16 and 2.49 ± 0.47 pg/mL SUPL vs. PL groups, respectively; Figure 4B). The level of serum TNF-α did not change during the study in the PL group (1.10 ± 0.17, 0.97 ± 0.18, 1.14 ± 0.20, and 1.03 ± 0.20 pg/mL for T1, T2, T3 and T4 time points, respectively; Figure 4C). In the SUPL group, we observed a reduction in the level of TNF-α after the rehabilitation program, as compared to the baseline level (1.32 ± 0.23, 1.11 ± 0.18, 1.07 ± 0.18, and 0.98 ± 0.15 pg/mL for T1, T2, T3 and T4 time points, respectively; Figure 4C). There were no changes in serum IL-10 concentration after supplementation, PLIF surgery, and the rehabilitation program in the PL, and the SUPL, as well as between groups (see Figure 4D).

## Discussion

One of the main findings of our study was that the serum concentration of vitamin D in LBP patients increased after 5 weeks of supplementation with a dose of 3200IU/day. We found that patients supplemented with vitamin D obtained the normal level of vitamin D. We observed that the serum concentration of vitamin D was slightly reduced in the SUPL group during next 14 weeks to subnormal levels; however, it was still significantly higher than before supplementation. Vitamin D supplementation is not a standard treatment in LBP patients, which condition is a major cause for sick leave in western countries. To the best of our knowledge, this study is the first to show that vitamin D supplementation improved recovery in this group of patients, an observation of significant socioeconomic impact. Simultaneously, the diminished levels of CRP, IL-6 and TNF-α, the pro-inflammatory markers, were detected in the SUPL group. The present study also showed that the subjective pain sensation decreased in both groups of patients following surgery and rehabilitation. However, a higher attenuation in the severity of pain was observed in the SUPL group after PLIF surgery and after rehabilitation, suggesting the positive effect of normalized vitamin D on the lowering pain sensation. Our data imply that preoperative supplementation with vitamin D was relevant and relieved the sensation of pain; therefore, it led to greater patient response during the recovery process. Recently, we showed that serum vitamin D deficiency was associated with paraspinal muscle atrophy and was conducive to mitochondrial dysfunction. When LBP patients were supplemented with vitamin D to suffice the serum vitamin D level, we found that muscle atrophy was attenuated and the mitochondrial function was increased (Dzik et al., 2019).

In the present study, LBP patients had serum vitamin D concentration before supplementation. These data are concordant with earlier reports, where vitamin D insufficiency was secondary to patients who were overweight, led a sedentary lifestyle, had inappropriate dietary habits, etc., (Martin and Reid, 2017; Pludowski et al., 2018). However, the major cause of vitamin D deficiency is related with underexposure to sunlight, which determines the synthesis of vitamin D. The optimal time for vitamin D synthesis in our latitude is between 11 am and 3 pm from the beginning of spring to mid-autumn. In Poland, vitamin D deficiency was found in 90% of adults, children and adolescents (Rusinska et al., 2018). Furthermore, Napiorkowska and coauthors showed that the concentration of vitamin D during wintertime was 13.5 ng/ml in women aged 60–90 (Napiorkowska et al., 2009).

An important finding of our study was that the serum concentration of vitamin D in LBP patients normalized after 5 weeks of supplementation with a daily dose of 3200 IU. We observed a mild reduction of the serum concentration of vitamin D during the following 14 weeks to subnormal level; however, this level was still significantly higher than before supplementation. It may be related to the half-life of vitamin D of 3–4 weeks (Zerwekh, 2008).

In the current study, the initial score of pain intensity in VAS was comparable in both groups. In the SUPL group, we observed that the level of vitamin D increased with a simultaneous reduction in pain severity. In contrast, such changes were not observed in the PL group. Our observations are in line with the data of Stoker and coauthors, who showed that patients with vitamin D deficiency awaiting spinal fusion experienced greater pain and obtained higher disability scores (Stoker et al., 2013). In another study on vitamin D level normalization, decreased pain intensity and improved muscular strength in vitamin D deficient immigrant women were achieved after 3 months of vitamin D and calcium supplementation (Englund et al., 2017).

The present study also showed that after surgery alone and postoperative rehabilitation subjective pain sensation significantly decreased in both groups of LBP patients. However, the attenuation of pain was higher in the SUPL group, which indicates the positive effect of normalized vitamin D level on pain relief during the recovery process. A greater reduction of pain intensity in the SUPL group could also be the result of better regeneration of the spine. Metzger and coworkers suggested that vitamin D dose-dependently increased the quality of bone after a spinal fusion procedure. Moreover, an adequate serum 25(OH)D_3_ level was associated with many health benefits and could be an important modifiable risk factor prior to fusion surgery (Metzger et al., 2015).

On the other hand, in both groups, LBP patients were overweight; therefore, this could also be a risk factor in the generation of pain, which is consistent with earlier reports (Okifuji and Hare, 2015; Martin and Reid, 2017). Since obesity aggravates chronic pain or presents a greater risk of having pain, one may speculate that weight loss should reduce pain. An early longitudinal observation study of approximately 800 women showed that the loss of 5 kg reduced the risk of developing painful gonarthrosis by 50%. Vincent and coauthors also observed a significant reduction in pain in the low back and knee, in the experimental group (bariatric surgery) after +/- 5% of body fat loss at the 3-month follow-up as compared with control subjects who did not undergo the surgery (Vincent et al., 2012). Moreover, obesity may contribute to the chronicity of back injury (Okifuji and Hare, 2015). Känel and coauthors suggested that low 25(OH)D_3_ levels are related to heightened central sensitivity (particularly augmented pain processing) upon mechanical stimulation in chronic pain patients (von Kanel et al., 2014). Possible mechanisms of vitamin D-dependent pain modulation include its anti-inflammatory effects mediated by reduced cytokine and prostaglandin release and direct inhibitory effects on T-cell production of cytokine IL-2, IL-17, IL-21. Furthermore, obesity may be associated with an elevated concentration of the immune system markers, for example, IL-6 (Bluher et al., 2005). However, in the current investigation, the body weight did not alter in both groups of LBP patients during the course. In patients with chronic widespread pain Koch and coworkers showed that the pro-inflammatory cytokines (IL-1b, IL-2, IL-6, IFN-γ, TNF-α) in the plasma correlate with pain intensity over a longer period of time (Koch et al., 2007). Therefore, it may be assumed that vitamin D supplementation had a beneficial influence on the reduction of pro-inflammatory markers in the SUPL group (T4), and therefore led to the relief of pain intensity. We did not find a positive correlation between pro-inflammatory markers and VAS combined with serum vitamin D concentration in the SUPL and the PL group; however, the SUPL patients showed a higher reduction of inflammation and attenuation of pain intensity as compared to the PL group.

In the present study, a significant decrease in the inflammation markers, CRP, IL-6, and TNF-α were detected in the SUPL group. The anti-inflammatory marker, IL-10, is able to suppress the production of pro-inflammatory cytokines. This cytokine was also linked with analgesia. In the review study, suppressed IL-10 function was observed in chronic pain patients (Milligan et al., 2012). Another animal study showed that after injury IL-10 was rapidly utilized and its level was insufficient to control pain and inflammation (Kwilasz et al., 2015). We did not find differences in the serum concentration of IL-10 between groups and time points. Our data are in line with Waterhouse and coworkers, who observed no effect of vitamin D supplementation on the serum IL-10 level (Waterhouse et al., 2015). However, in another study, supplementation with vitamin D positively affected the serum concentration of IL-10 (Schleithoff et al., 2006). Possible explanations for these discrepancies may include small sample sizes that were too small, an insufficient duration or dose of supplementation to elicit a response, or other unknown factors in studied LBP patients. Moreover, vitamin D deficiency may act as an aggravating factor of dysregulated inflammation and activation in the immune system and, in this way, can be viewed as a modifiable risk factor responsible for increased inflammation. Although we did not measure the whole spectrum of cytokines, our data are sufficient to assume the beneficial effect of combined vitamin D supplementation and early rehabilitation on the immune system in LBP patients undergoing surgery.

Importantly, there were no significant differences between groups regarding medication. All the patients used only perioperative antibiotics, postoperative NSAIDs, perfalgan and tramadol, which are among the most common pain relief medicines. All LBP patients got the same treatment for the same time duration. Thus, we presumed that this treatment did not have an effect on pro and anti-observed inflammatory markers differences in both the groups of LBP patients.

**FIGURE 5:**
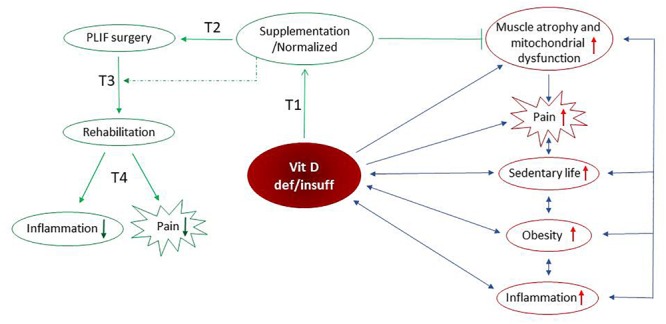
Possible mechanism(s) of vitamin D-dependent pain modulation include its anti-inflammatory effects mediated by the reduced cytokine(s). The sedentary lifestyle, obesity, elevated inflammation as well as paraspinal muscle atrophy and mitochondrial dysfunction (Dzik et al., 2018) reversely, are associated with vitamin D deficiency/insufficiency. We assume that above-listed factors intensify the pain in LBP patients. Preoperative vitamin D supplementation is accompanied by normalized serum vitamin D concentration, inhibition of muscle atrophy and restoration of mitochondrial function. After supplementation with vitamin D, the reduction of inflammation and alleviation of pain is observed in LBP patients after PLIF surgery followed by early rehabilitation. Taken together, our study support anti-inflammatory and pain-relieving actions of vitamin D that may promote the recovery process. ↓ – decrease of factor, ↑ – increase of factor, ⊣ – inhibition, blue arrows – negative interaction, green arrow – positive interaction/protocol time.

Recently published studies showed that supplementation with vitamin D had a beneficial effect on the paraspinal muscle weakness and oxidative stress (Bang et al., 2018; Dzik et al., 2018). The protein content of VDR in skeletal muscle is dependent on serum vitamin D level. Our group showed a positive correlation between the serum vitamin D concentration and the content of VDR in the paraspinal muscle of the supplemented group of LBP patients (Dzik et al., 2018). Taken together, the lower markers of lipid and protein peroxidation (Dzik et al., 2018) attenuated atrophy in the paraspinal muscle (Bang et al., 2018), combined with decreased serum CRP, IL-6 and TNF-α levels and reduced the VAS score in the current study, suggesting that LBP patients supplemented with vitamin D have a better and faster possibility of recovery with reduced pain intensity. Moreover, our results suggest that vitamin D supplementation could have a role in the management of chronic pain (Figure 5).

## Limitation of the Study

A potential limitation of this study was a small group of respondents. Furthermore, we did not monitor the patients’ diets and other components in both groups. Another limitation of the study was the examination of only four markers of inflammation status.

## Conclusion

Our data demonstrate that supplementation with vitamin D enhanced the reduction of systemic inflammation markers, and when combined with surgery and early postsurgical rehabilitation, it may decrease the intensity of pain in LBP patients undergoing PLIF. The study supports the anti-inflammatory and pain-relieving actions of vitamin D that may promote the recovery process. Thus, we propose that LBP patients undergoing spine surgery should use vitamin D as a supplement not only before but also after surgery due to its positive effect.

## Ethics Statement

The study was approved by the local institutional Bioethical Committee in Gdansk (No. NKBBN/120/2012) and conformed to the Declaration of Helsinki and was registered as a Clinical Trial NCT03 417700 https://clinicaltrials.gov.

## Author Contributions

KK and JK: conceptualization and writing – original draft. JK: data curation and project administration. KK, WS, PS, KD, and DF: formal analysis. KK, WS, EL PS, DF, WL, and JK: investigation. WS, WK, and JK: methodology. WS and JK: supervision. WS, EL, PS, KD, DF, WK, and WL: writing – review and editing.

## Conflict of Interest Statement

The authors declare that the research was conducted in the absence of any commercial or financial relationships that could be construed as a potential conflict of interest.
